# Immediate Versus Late Flap Coverage for Traumatic Soft Tissue Defects of Lower Extremity: A Comparative Observational Study

**DOI:** 10.7759/cureus.22800

**Published:** 2022-03-03

**Authors:** Vishal Patil, Bhaskar Sarkar, Mohd Altaf Mir, Quamar Azam, Madhur Uniyal, Ajay Kumar, Vishal Mago, Nilesh Jagne, Divakar Goyal, Rajesh Maurya

**Affiliations:** 1 Trauma Surgery and Critical care, All India Institute of Medical Sciences, Rishikesh, Rishikesh, IND; 2 Trauma Surgery and Critical Care, All India Institute of Medical Sciences, Rishikesh, Rishikesh, IND; 3 Burns and Plastic Surgery, All India Institute of Medical Sciences, Bathinda, Bathinda, IND; 4 Burns and Plastic Surgery, All India Institute of Medical Sciences, Rishikesh, Rishikesh, IND

**Keywords:** late reconstruction, lower limb trauma, immediate reconstruction, flap coverage, extremity injury

## Abstract

Background

The lower extremity trauma in patients often gets operated on late for the wound coverage and is associated with more health costs and other resources. Therefore, this study has been conducted to compare the outcome in terms of flap survival, complication rates, and hospital stay between immediate and late flap coverage of lower extremity traumatic wounds.

Methods

The comparative analysis of outcome is done in terms of flap survival, complication rates, and hospital stay after immediate and late flap coverage of 25 (n = 25) patients of lower extremity traumatic wounds in each group. The patients were observed, and data obtained were tabulated in a Microsoft Excel spreadsheet. The statistical analysis was done using IBM SPSS (V26.0, IBM Corporation, Armonk, NY, USA) statistical software. The chi-square test was used for descriptive data and the student's unpaired t-test for discrete-continuous data analysis. The p-value of less than 0.05 is considered significant.

Results

The mean defect size with SD in the immediate flap cover group is 54.5 ± 29.5 cm2, while in the late flap cover group, it is 85 ± 65 cm2 with a significant p-value of 0.0378. The mean flap size with SD in the immediate flap coverage group is 70.5 ± 34.5 cm2, while in the late flap coverage group, it is 117 ± 87.5 cm2, and the difference is statistically significant. The mean hospital stay with SD in the immediate flap coverage group is 7.5 ± 2.5 days. In contrast, in the late flap coverage group, it is 29.5 ± 8.5 days, and the difference is statistically very significant.

Conclusion

There are equivalent results in patients undergoing immediate and late flap coverage for the traumatic soft tissue defects of the lower extremity. There is a significant decrease in the hospital stay after immediate flap reconstruction, which subsequently reduces both direct and indirect health costs. However, there is a larger size flap requirement in cases of immediate lower extremity wound coverage.

## Introduction

Trauma is the primary cause of death and disability in developing countries and is responsible for more years of life lost than most human diseases [1]. A road traffic accident is the most common cause of trauma (45-50%), and the most commonly affected age group is 11-40 years (60-65%) with a predominance of males (75-80%) [2]. Road traffic accidents, falling from a height, sports injuries, and firearm injuries are the common mechanism of injury for traumatic soft tissue defects of the lower extremity. The most common injury is bony injury (60-67%), followed by soft-tissue injury (30-35%) in lower limbs [2].

Treatment of extremities has evolved over the last decade. Nowadays, many extremities that would have required amputation are salvaged. The successful salvage of a limb depends on the proper application of ortho-plastic and plastic surgical principles, including adequate debridement, fracture stabilization, and soft tissue coverage [3-5]. The treatment goal remains extremity salvage, and these traumatic injuries carry a high potential for morbidity and secondary amputation rates up to 10 to 15% [6]. Gustilo grade IIIB and IIIC fractures require complex soft-tissue coverage and are associated with higher rates of infection, non-union, prolonged hospitalization, time lost from work, and increased healthcare-related costs [5-7]. Flap coverage is preferred in cases where secondary reconstruction is anticipated; flexor joints are exposed (to avoid contractures), nerves and vessels are exposed, presence of dead space, and when the durability of transferred tissue is required [8]. In severe lower extremity injury, immediate soft-tissue coverage within 72 hours is associated with more favorable outcomes in terms of length of hospitalization and prevention of infection. It has the lowest complication rate and highest success rate [9]. It minimizes the risk of increasing bacterial colonization, helps prevent infection, and facilitates bone union. Management of extremity trauma with bone and soft tissue injury remains a formidable problem. The lower extremity trauma in patients often gets operated on late for the wound coverage and is associated with the utilization of more health costs and other resources. This study has been conducted to compare the outcome in terms of flap survival, complication rates, and hospital stay between immediate and late flap coverage of lower extremity traumatic wounds.

## Materials and methods

Study design

This is a prospective comparative observational study of immediate and late flap coverage for traumatic soft tissue defects of the lower extremity. The study is conducted at our institution, which is one of the apex trauma centers of north India. The study has been done after institutional ethical committee approval (IEC), and the registration vide reference number AIIMS/IEC/21/34.

Study population

**Figure 1:**
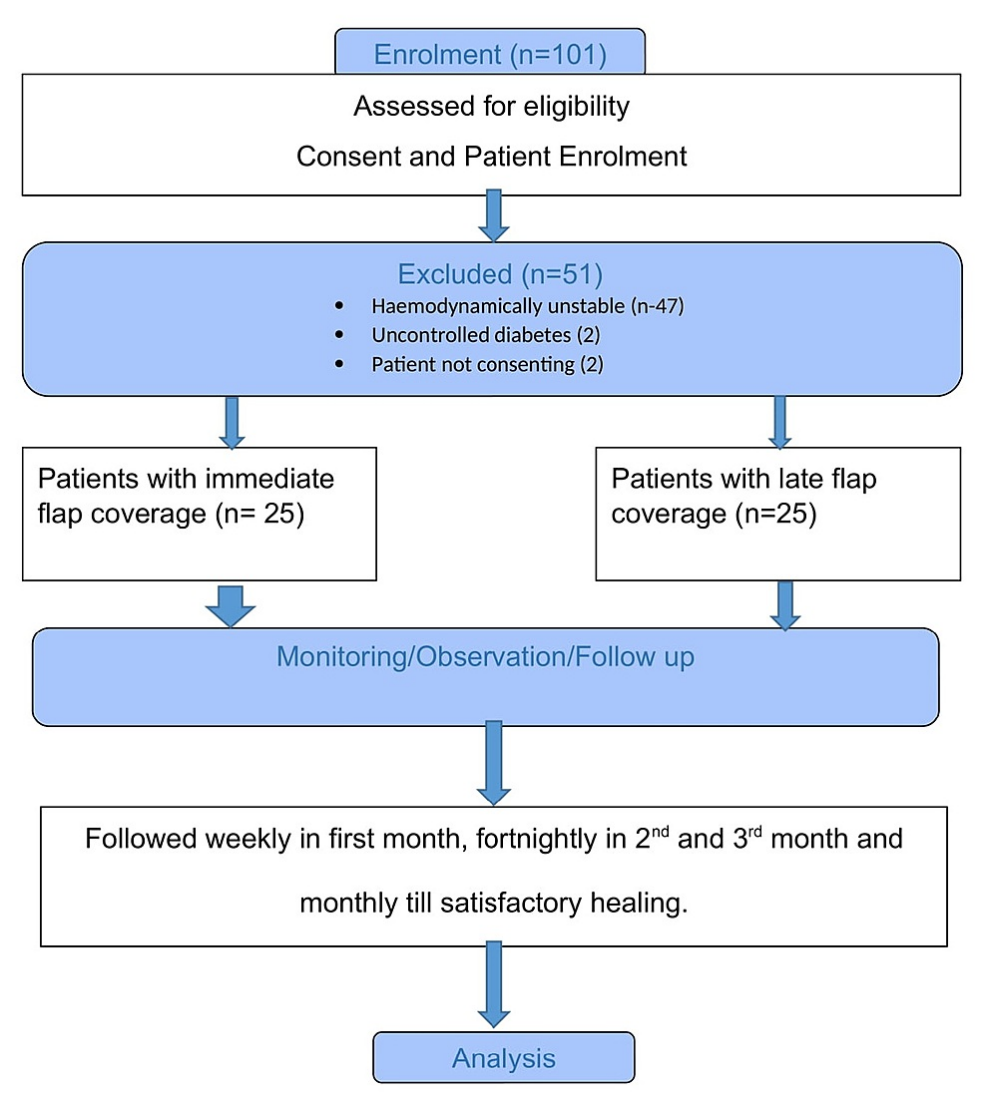
Consort study design diagram. This flow chart depicts the recruitment of eligible patients and their allocation in immediate and late flap coverage groups after excluding patients who do not fit for the study and subsequent observation, follow-up, and analysis.

Patients with traumatic soft-tissue defect of the lower extremity with associated bony injury, who underwent immediate flap coverage within 72 hours (n = 25) and late flap coverage anytime then after (n = 25) were included in the study and observed prospectively for hematoma, seroma formation, surgical site infection, flap failure and success, flap congestion, and hospital stay. The study design and population are described in Figure 1.

Sample size

Data from a previous retrospective study by Zhou S et al. [10] were used to calculate the sample size. The sample size calculation was performed using OpenEpi (OpenEpi, Version 3, open-source calculator-SS Cohort) online tool, which is based on the formula described by Kelsey JL et al. [11]. Considering 80% power with 5% significance and a high effect size, the sample size calculated is 25 in each group.

Selection criteria for study participants

All traumatic patients of both sexes from 16 to 59 years of age having Gustilo grade IIIB and IIIC fractures with soft-tissue defect of lower extremity requiring flap coverage were included in the study. Children of less than 16 years and elderly of more than 59 years of age, hemodynamically unstable and critically ill patients not fit for surgery, uncontrolled diabetes, known case of peripheral vascular disease, immunocompromised patients, and patients not consenting for surgery were excluded.

Patient evaluation

The patient evaluation was done in accordance with advanced trauma life support (ATLS) guidelines viz. triage, a primary survey of trauma, a secondary survey of limb trauma, a history of mechanism, time of injury, assessment of the wound, limb vascularity, and fracture pattern.

A careful history was taken looking into the etiological factors and type of treatment at the time of injury. Each patient was subjected to a thorough clinical examination to assess the wound's status and rule out the involvement of various other systems. All patients have undergone baseline investigations like hemogram, renal and liver function test, random blood sugar estimation, chest radiograph, radiograph of the injured part, and electrocardiogram. Computerized tomographic angiography of extremity was done when the vascular injury was suspected. Swabs from wounds were sent for culture and sensitivity in those who presented any time in the course of hospitalization with the signs and symptoms of local wound infection in both groups.

Flap coverage of the wound

Defects of the lower extremity were classified into upper third leg defects, middle third leg defects, and lower third leg defects for ease of discussion. Once the decision of surgical closure of a wound has been made, appropriate debridement was undertaken before a final coverage option was chosen. If the wound is suitable for the flap cover, it has been covered with a muscle or fascio-cutaneous flap within 72 hours in case of immediate flap coverage group and any time after 72 hours in late flap coverage group.

Flap observation and follow-up

Handheld Doppler with an 8 MHz vascular probe was used to plan and design a flap for soft tissue defect reconstruction and assess its vascularity following reconstruction. The versatility of flaps was assessed in terms of flap failure and success rate. In our study, we defined flap failure as loss of more than 50% of the flap, flap success as uptake of more than 95% of the flap, and margin necrosis within 2 cm of distal margin of the flap. Further dressings were done till the flap inset line was healthy and clinically observed for healing and any complications. All patients were followed weekly in the first month, fortnightly in the second and third months, and then monthly until the healing was satisfactory.

Data collection and statistical analysis

The data were recorded as per set performa, which was then tabulated in the Microsoft Excel spreadsheet. The data were analyzed using statistical software (IBM SPSS V26.0, IBM Corporation, Armonk, NY, USA). The results were expressed as mean ± SD for discrete data and proportions for descriptive data. Descriptive data were analyzed by the Chi-squared test and discrete data using unpaired student's t-test between the two groups. Statistical significance is set at 5% (p <0.05). Table 1 shows the summary of cases and statistical comparison of the data of the two groups.

## Results

**Figure 2:**
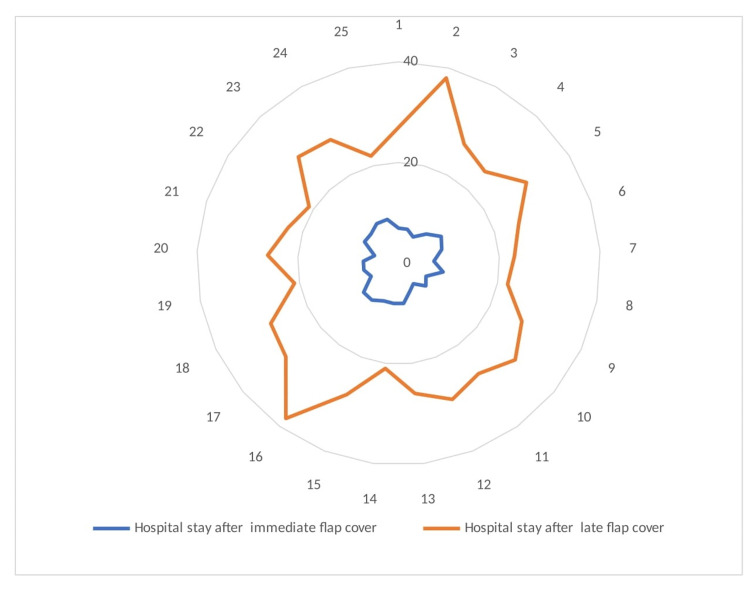
Radar chart with markers of comparison of hospital stay. It depicts the sizable significant gap of hospital stay between the immediate and late flap coverage groups. Furthermore, it shows that most of the patients of the immediate flap coverage group were discharged from the hospital within 10 days, while in case of late coverage, patients were discharged after 20 days from the hospital.

In our study, all the injuries to the lower extremities were due to road traffic accidents in both groups. The young males (more than 80%) of 37-38 years were mostly injured in both groups. The middle 1/3rd of the right leg was the most common injury site of the lower extremity in each group. The age, sex, mode of injury, limb laterality, and location of injury at extremity were statistically comparable with the p-value of more than 0.05. There were equivalent results in terms of flap outcome in patients undergoing immediate and late flap coverage for the traumatic soft-tissue defects of the lower extremity. The mean defect size with SD in the immediate flap cover group was 54.5 ± 29.5 cm2, while in the late flap cover group, it was 85 ± 65 cm2 with a significant p-value of 0.0378. The mean flap size with SD in the immediate flap cover group was 70.5± 34.5 cm2, while in the late flap cover group, it was 117± 87.5 cm2 with a p-value of 0.0170. The mean hospital stay with SD in the immediate flap cover group was 7.5 ± 2.5 days, while in the late flap cover group, it was 29.5 ± 8.5 days with a p-value of < 0.0001 (Figure 2).

The fascio-cutaneous flaps were more commonly used for immediate coverage and muscle flaps for late coverage, but the difference was insignificant between the groups. The drain was used in all immediate reconstructions and never in the late reconstruction group. Pre-reconstruction non-surgical aids were used in most of the late reconstruction group cases and never in the immediate reconstruction group. Conventional dressing in 72% and vacuum-assisted closure (VAC) in 28% with a statistically significant difference were the non-surgical aids used in the late reconstruction group, increasing the treatment costs. The flap survival and complications, including the flap necrosis, were comparable in both groups. The summary of cases and statistical comparison of the data of two groups is depicted in Table 1.

**Table 1:** Summary of cases and their statistical comparison. RTI: Road traffic injury; SB: Superiorly based; IB: Inferiorly based; PTA: Posterior tibial artery; FC: Fascio-cutaneous; PF: Perforator flap; PA: Peroneal artery; RSF: Reverse sural artery flap; GMF: Gastrocnemius flap; VAC: Vacuum-assisted closure; IS: Insignificant; S: Significant.

Characteristics	Parameters	Immediate flap coverage (n=25)	Late flap coverage (n=25)	Statistics
Age of patients	(Mean ± SD) years	37.5 ± 21.5	38 ± 22	t = 0.081, p = 0.9356
Sex	Male (n) %	(22) 88	(23) 92	Χ^2 ^= 0.218, p = 0.6407
	Females (n) %	(3) 12	(2) 8	Χ^2 ^= 0.050, p = 0.8226
Mode of injury	RTI (n) %	(25) 100	(25) 100	-
Laterality	Right leg (n) %	(14) 56	(14) 56	Χ^2 ^= 0.000, p = 1.0000
	Left leg (n) %	(11) 44	(11) 44	Χ^2 ^= 0.000, p = 1.0000
Thirds of leg	Proximal (n) %	(7) 28	(6) 24	Χ^2 ^= 0.102, p = 0.7496
	Middle (n) %	(11) 44	(10) 40	Χ^2 ^= 0.080, p = 0.7767
	Distal (n) %	(7) 28	(9) 36	Χ^2 ^= 0.360, p = 0.5483
Defect size	(Mean ± SD) cm^2^	54.5 ± 29.5	85 ± 65	t = 2.136, p = 0.0378
Flap	SB, PTA, FCPF (n) %	(3) 12	(2) 8	Χ^2 ^= 0.050, p = 0.8226
	IB, PTA, FCPF (n) %	(12) 48	(8) 32	Χ^2 ^= 1.307, p = 0.2530
	IB, PA, FCPF (n) %	(1) 4	(2) 8	Χ^2 ^= 0.348, p = 0.5555
	RSF (n) %	(4) 16	(6) 24	Χ^2 ^= 0.490, p = 0.4839
	GMF (n) %	(5) 20	(7) 28	Χ^2 ^= 0.430, p = 0.5121
Adjuvant therapy	Conventional dressing (n) %	(0) 0	(18) 72	Χ^2 ^= 27.563, p = < 0.0001
	VAC (n) %	(0) 0	(7) 28	Χ^2 ^= 7.977, p = 0.0047
Flap size	(Mean ± SD) cm^2^	70.5 ± 34.5	117 ± 87.5	t = 2.472, p = 0.0170
Flap survival	Yes (n) %	25) 100	25) 100	-
	No (n) %	(0) 0	(0) 0	-
Flap necrosis	(Mean ± SD) cm	1.5 ± 0.5	1.5 ± 0.5	t = 0.000, p = 1.0000
Hospital stays	(Mean ± SD) days	7.5 ± 2.5	29.5 ± 8.5	t = 12.415, p < 0.0001
Complications	Yes (n) %	(7) 28	(5) 20	Χ^2^ = 0.430, p = 0.5121
	No (n) %	(18) 72	(20) 80	Χ^2^ = 0.430, p = 0.5121
Different complications	Surgical site infection (n) %	(3) 12	(3) 12	Χ^2 ^= 0.000, p = 1
	Flap congestion (n) %	(2) 8	(2) 8	Χ^2 ^= 0.000, p = 1
	Seroma (n) %	(1) 4	(1) 4	Χ^2 ^= 0.000, p = 1
	Margin necrosis	(6) 24	(5) 20	Χ^2 ^= 0.114, p = 0.7354
	Flap loss (n) %	(0) 0	(0) 0	-
	Osteomyelitis (n) %	(0) 0	(0) 0	-
	Donor site morbidity (n) %	(0) 0	(0) 0	-

**Figure 3:**
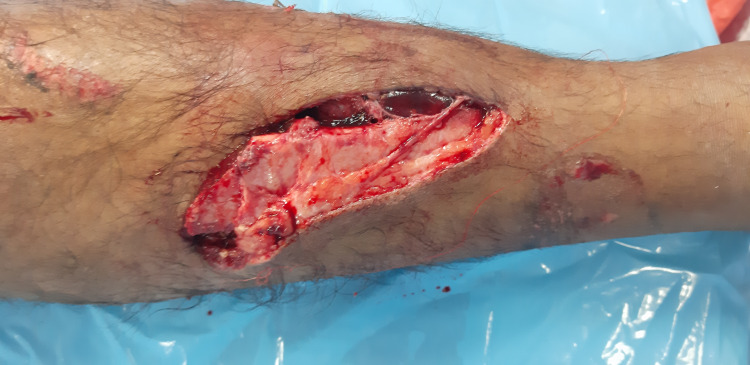
Preoperative photograph. It depicts the 12x6 cm traumatic soft tissue defect and Gustilo type IIIB tibial fracture of the middle 1/3rd of the left leg within 2 hours of trauma.

Here we describe a representative case of immediate flap coverage after lower extremity injury. A young male patient sustained a road traffic accident, and his left lower leg got injured, having extensive soft tissue injury and Gustilo type III B tibial fracture (Figure 3).

**Figure 4:**
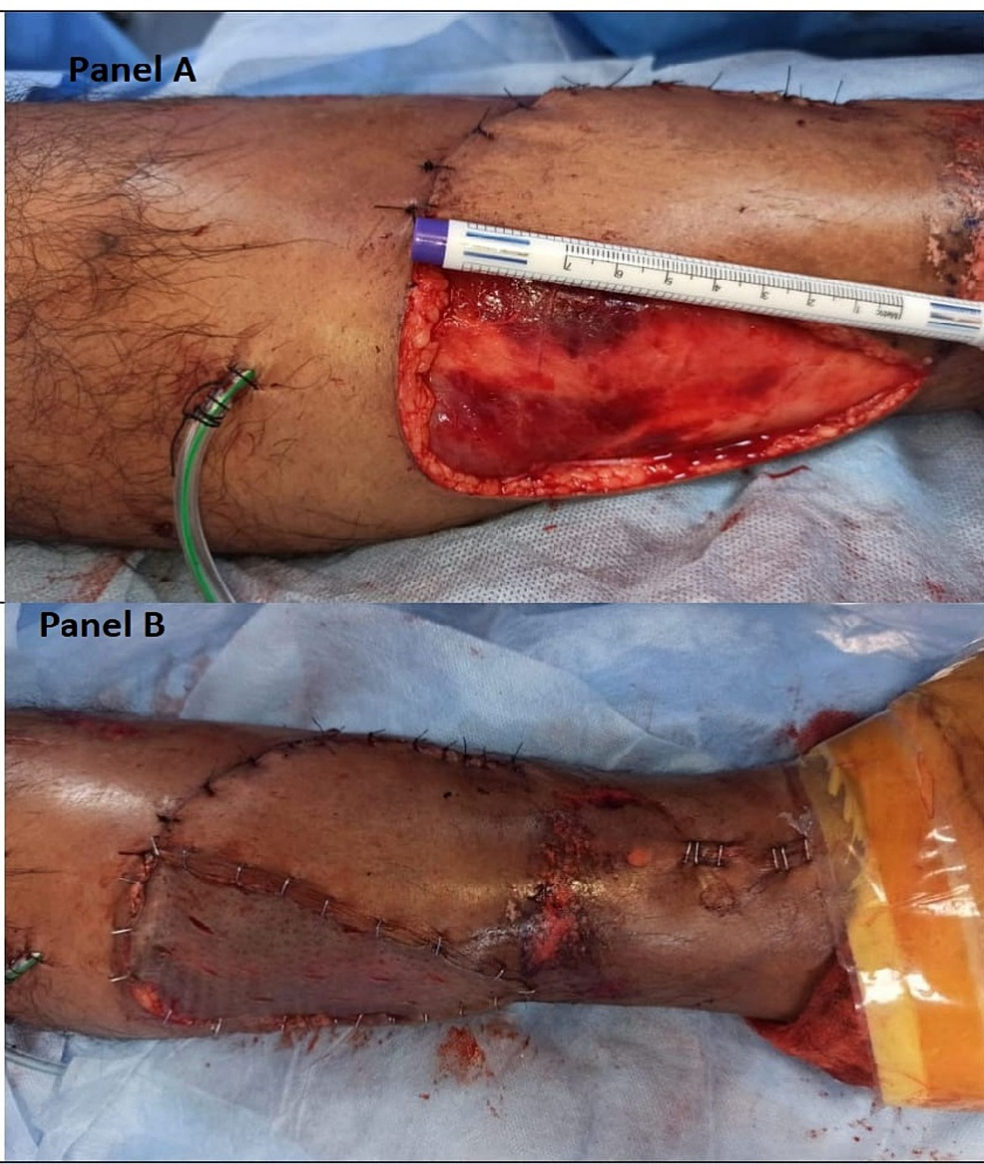
Intraoperative photograph. Panel A shows the immediate flap coverage of the traumatic wound of the middle 1/3rd of the left leg with an inferiorly based posterior tibial artery perforator flap. It also depicts the raw area at the donor site. Panel B depicts the immediate flap coverage of the traumatic wound of the middle 1/3rd of the left leg with an inferiorly based posterior tibial artery perforator flap and skin grafting of the donor site.

After tibial intramedullary nailing, the soft tissue coverage was achieved with inferiorly based posterior tibial perforator flap (Figure 4).

**Figure 5:**
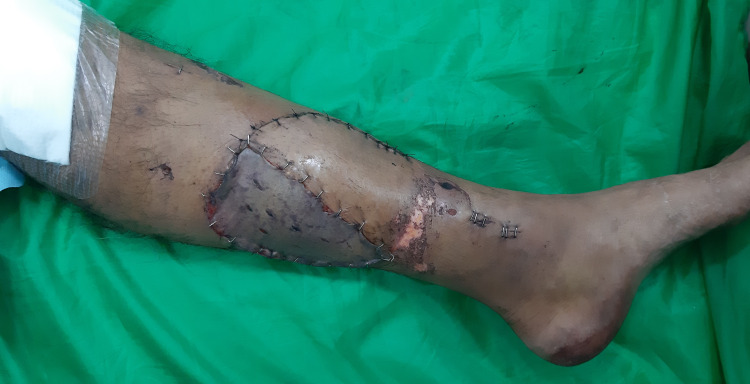
Postoperative photograph. It shows the healthy vascular flap and good take of donor site skin grafting at postoperative day 7.

The patient was discharged within 10 days from the hospital after healthy healing of the flap (Figure 5).

**Figure 6:**
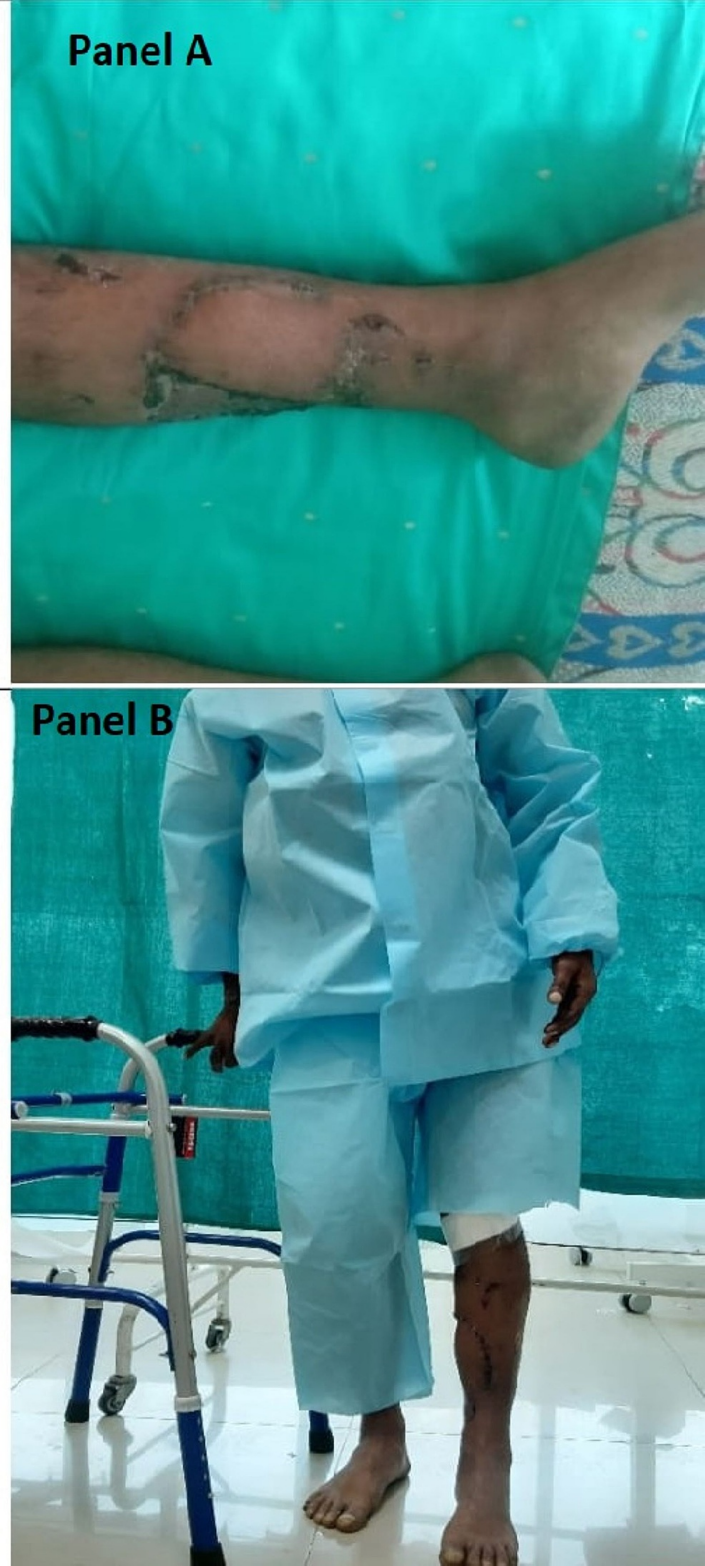
Follow-up photograph. Panel A shows the healed flap and donor site skin grafting at four weeks of follow-up, and panel B depicts that patient can walk with a support of a walker (good weight-bearing) at eight weeks.

Flap and bone healing was uneventful (Figure 6, Panel A), and the patient could bear some amount of weight (Figure 6, Panel B). 

**Figure 7:**
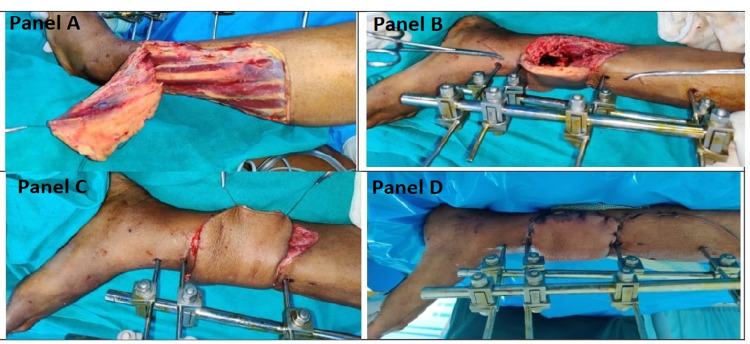
Case of late flap coverage. Panels A-C are intraoperative photographs, and panel D is a postoperative photograph. Panel A depicts the elevation of an inferiorly based peroneal artery perforator flap, panel B shows the transfer of flap on the wound on the lower third medial aspect of the left leg, panel C depicts complete coverage, and panel D shows complete healing at two weeks.

In another case, a 25-year-old male discussed here has sustained a road traffic accident with tibial fracture and extensive soft tissue loss on the medial aspect of the lower third of the left leg. The reconstruction with a flap is described in Figure 7. 

## Discussion

The lower extremity injuries, if not managed timely, lead to complications. The successful salvage of a limb depends on the proper application of ortho-plastic surgical principles, including adequate debridement, fracture stabilization, and soft tissue coverage [3-5].

There are only a few prospective and case-control studies comparing immediate and delayed flap coverage of lower extremity traumatic soft-tissue defects, which have suggested improved outcomes in terms of flap success rate, decreased infection rate, and lesser hospital stay. Meyer C et al., in their study conducted from 1997 until 2003, treated 36 patients using early neurovascular flap coverage. In their study, the flaps healed without functional impairment in 35 cases. Primary healing was achieved in 30 patients. Five cases had partial loss of the skin island, whereby subcutaneous tissue remained vital. In their study, revision by mesh-graft transplantation led to successful healing in these patients. The authors have observed flap necrosis in one patient only. Neurovascular flaps usually result in reliable and complete healing of lower extremity soft-tissue defects [12]. Byrd HS et al. performed a study in which 18 patients with lower extremity wounds with underlying bone fractures were treated employing a combined ortho and plastic surgical approach based on the tenets of early radical debridement, a second look operation, muscle or musculocutaneous flap cover within five days of injury, external pin fixation, and ambulation within the first three weeks of injury. They found that all fractures united in a mean time of 4 months. The mean hospitalization in their patients was 4.2 weeks. There has been no chronic infection, osteomyelitis, non-union, shortening, or tissue breakdown [13]. Godina M studied 532 patients who underwent microsurgical reconstruction following trauma to their extremities. They divided the reconstruction cases into three groups for the purpose of the review. Group 1 underwent free-flap transfer within 72 hours of the injury, group 2 between 72 hours and three months of the injury, and group 3 between three months and 12.6 years, with a mean of 3.4 years. They analyzed their results with respect to flap failure, infection, bone-healing time, length of hospital stays, and the number of operative procedures. The flap failure rate in their study was 0.75% in group 1, 12% in group 2, and 9.5% in group 3 (p < 0.0005 early versus delayed; p < 0.0025 early versus late). They also found that postoperative infection occurred in 1.5% of group 1, 17.5% of group 2, and 6% of group 3. Bone-healing time was 6.8 months in group 1, 12.3 months in group 2, and 29 months in group 3. The average length of total hospital stay was 27 days for group 1, 130 days for group 2, and 256 days for group 3. The number of operations averaged 1.3 for group 1, 4.1 for group 2, and 7.8 for group 3 [4].

Chua W et al. performed a study between 2000 to 2009 by reviewing medical records of 83 men and six women with a mean age of 38 years who underwent fixation for open tibial fractures of Gustilo grades IIIB and IIIC followed by flap coverage within 72 hours (n = 30) and after 72 hours (n = 59). In their cases, all fractures were treated within 24 hours, and outcome measures included bone union, infection, flap failure, and the need for secondary procedures to achieve union. They compared the early and late flap coverage groups. They found that the early flap coverage was associated with a shorter length of hospitalization (31.4 vs. 55.8 days, p <0.01), lower deep infection rates (23% vs. 54%, p <0.01), and a smaller number of surgical procedures (6.4 vs. 9.2, p = 0.01). They found that the two groups did not differ significantly in terms of the time to bone union, flap failure, amputation, and the need of secondary procedures to facilitate bone union. They concluded that in cases of severe open tibial fractures, early soft-tissue coverage (within 72 hours) was associated with more favorable outcomes in terms of length of hospitalization and infection [10,14]. We also found that the mean hospital stay is less after immediate flap reconstruction than after late reconstruction (Figure 2).

However, in our study, the outcome and complications are equivalent after immediate and late flap coverage of lower extremity traumatic wounds. In addition, the hospital stay in our study is lesser after immediate flap coverage than after late flap coverage, as is true with other studies [4,10-13]. However, many of these studies have suggested further comparative prospective observational studies to achieve a higher level of evidence. Therefore, our study is a comparative, prospective, and observational study to assess the outcome after the immediate and late flap coverage of lower extremity wounds in order to augment the available evidence.

Our study observed that road traffic injuries (100%) are the most frequent cause of lower extremity trauma in young adult males (more than 80%) of 37-38 years of age. We also observed that the mid 1/3rd of the right leg is the most commonly injured site of the lower extremity. Similar reports have been published before [10], which also suggest that road traffic accident is the most common cause of trauma (45-50%) and the most commonly affected age group is 11-40 years (60-65%) with a predominance of males (75-80%). We used fascio-cutaneous flaps more commonly for immediate coverage and muscle flaps for late coverage; however, the difference is insignificant statistically between the groups. Similar results are also reported by Meyer C et al. [12]. In all immediate reconstructions, the drains were used to avoid hematoma or seroma formation and were never used in the late reconstruction. Pre-reconstruction non-surgical aids like conventional dressings (72%) and VAC (28%) were used in most of the cases of the late reconstruction group and never in the immediate reconstruction group. The flap survival and complications, including flap necrosis, are equivalent in both groups. There is no significant difference of means and proportions of all the outcome parameters except the hospital stay. The patients were usually discharged on postoperative day 7 after immediate reconstruction and postoperative day 20 after late reconstruction of lower extremity soft-tissue defects. However, there is a significant statistical difference of mean hospital stay between the two groups, which signifies that immediate flap coverage of traumatic wounds decreases hospital stay and reduces direct and indirect healthcare costs. In our study, we observed that the defect size of early post-traumatic wound is larger than the late post-traumatic wound, the reason being that primary contraction of peri-wound tissue leads larger size of wound after immediate trauma while a secondary wound contraction is a normal healing process that results in smaller wounds after few days of trauma. This is the reason for the larger size flap requirement for the immediate post-traumatic wound coverage than late wound coverage.

The limitation of our study is the smaller sample size hence needs further studies with larger samples or multicentric studies. Another limitation is that our study is not a randomized controlled trial; hence it is suggested to conduct further evaluation through randomized controlled study to augment and strengthen the level of evidence.

## Conclusions

This study concluded that there is a significant decrease in hospital stay after immediate flap reconstruction of lower extremity traumatic soft-tissue defects without fear of any grievous complications. However, other outcome measures, including flap survival, are equivalent in patients undergoing immediate and late flap coverage for the traumatic soft-tissue defects of the lower extremity. This subsequently reduces both direct and indirect healthcare costs. Our study is a landmark study and will be the basis for further research, especially randomized controlled trials, to augment the level of evidence.
